# Folic acid supplementation, preconception body mass index, and preterm delivery: findings from the preconception cohort data in a Chinese rural population

**DOI:** 10.1186/s12884-015-0766-y

**Published:** 2015-12-15

**Authors:** Yuanyuan Wang, Zongfu Cao, Zuoqi Peng, Xiaona Xin, Ya Zhang, Ying Yang, Yuan He, Jihong Xu, Xu Ma

**Affiliations:** National Research Institute for Family Planning, No. 12, Dahuisi Road, Haidian District, Beijing, 100081 China; Graduate School of Peking Union Medical College, No. 9, Dongdansantiao, Dongcheng District, Beijing, 100730 China

**Keywords:** Folic acid supplement, Maternal body mass index, Interaction, Preterm deivery, Preconception

## Abstract

**Background:**

Folic acid (FA) supplementation before and during the first trimester can reduce the risk of occurrence of preterm delivery (PTD). Preconception body mass index (BMI) is also associated with PTD. This study aimed to investigate the combined effect of FA supplements and preconception BMI on the risk of PTD.

**Methods:**

The data of a cohort from 2010–2011 that was obtained through a preconception care service in China was used (including 172,206 women). A multivariable regression model was used to investigate the association between maternal preconception conditions and the risk of PTD. The interaction of preconception BMI and FA supplementation was measured by a logistic regression model.

**Results:**

Taking FA supplements in the preconception period or in the first trimester reduced the risk of PTD (odds ratio [OR] = 0.58 and OR = 0.61, respectively). Women with an abnormal BMI had an increased risk of PTD (OR = 1.09, OR = 1.10, and OR = 1.17 for underweight, overweight, and obese, respectively). Preconception BMI showed an interaction with the protective effect of FA supplementation for PTD. With regard to the interaction of FA supplementation, the adjusted odds ratio (aOR) was 0.57 (95 % CI: 0.51, 0.64) in underweight women, 0.85 (95 % CI: 0.73, 0.98) in overweight women, and 0.77 (95 % CI, 0.65, 0.91) in obese women. Preconception BMI also showed an interaction with the time of FA supplementation. Women with a normal BMI who began to take FA supplements in the preconception period had the lowest risk of PTD (aORs: 0.58 vs. 0.65 beginning in the first trimester). The aORs at preconception and the first trimester in the underweight group were 0.56 vs. 0.60. The aORs at preconception and the first trimester were 0.94 vs. 0.65 and 1.15 vs. 0.60 in the overweight and obesity groups, respectively.

**Conclusions:**

In our study, FA supplements reduced the risk of PTD, while abnormal BMI raised the risk of PTD, although higher BMI categories did not have this higher risk once adjusted analysis was conducted. The protective effect of FA supplementation for PTD was reduced in women with overweight or obesity. To get better protection of FA supplementation, women with normal BMI or underweight should begin to use in preconception, while women with overweight or obesity should begin to use after conception.

## Background

Preterm delivery (PTD) is defined as neonates who are born alive before 37 weeks of pregnancy are completed. PTD is the second largest direct cause of child deaths in children younger than 5 years and causes approximately 1 million deaths annually worldwide. Additionally, PTD causes lifelong problems for many survivors [1].

The incidence of PTD was 7.1 % in a multicenter, hospital-based investigation (based on 107,905 deliveries) in China. The proportion of PTD among the causes of neonatal death has significantly increased from 33.6 % in 2003 to 40.9 % in 2008. PTD is the leading cause of neonatal death in China [2]. Because of the large population in China, China has become the second largest country with annual preterm deliveries. PTD is a serious public health problem in China.

PTD is thought to be a syndrome that is initiated by multiple mechanisms. However, the precise mechanism of PTD has not been established. Therefore, factors associated with preterm birth, but not obviously in the causal pathway, have been sought to explain preterm labor.

Folic acid (FA), an oxidized synthetic water-soluble member of the vitamin-B complex family, plays an essential role in one-carbon metabolism. The clinical application of FA supplementation to prevent neural tube defects (NTD) had been well proven in the last 20–25 years. Pregnant women are at risk of folate deficiency because pregnancy greatly increases folate requirements, especially during periods of rapid fetal growth (i.e., in the second and third trimesters). Although the relation between maternal folate status with NTD has been well established [3, 4], the association of folate status with other adverse pregnancy outcomes is still unclear [5]. Recently, some researchers have investigated maternal FA supplementation and PTD, but the findings were largely inconclusive. Some results supported protective effects [6–9] and some did not [10–14, 15]. A large population-based cohort study conducted in China showed that daily intake of 0.4 mg folic acid reduced the risk of PTD [6]. A cohort in Singapore showed that higher plasma folate concentrations were associated with a longer gestational age and tended to be associated with a lower risk of PTD [16]. Similar results were obtained in some prospective observational studies in the USA [17, 18]. Some researches have shown that maternal FA supplementation during pregnancy protects against lipopolysaccharide-induced PTD through its anti-inflammatory effects [19–21]. Researchers have hypothesized that lower maternal serum folate concentrations during pregnancy is associated with higher maternal plasma homocysteine concentrations, which cause pre-eclampsia and affect placental function, eventually leading to PTD [22, 23].

Abnormal preconception body mass index (BMI) is a risk factor for PTD [24, 25, 26]. The spontaneous PTD rate is higher in pre-pregnancy underweight women (BMI <18.5 kg/m^2^) than in normal-weight women [27, 28]. Some researchers have found that indicated PTD and premature rupture of the membranes are associated with preconception obesity, especially in extremely obese groups [27, 29]. Obese women are more likely to develop pre-eclampsia and diabetes, and have indicated preterm births associated with these disorders [30]. In a Boston birth cohort, pre-pregnancy obesity was associated with a decreased odds of PTD (0.76) and underweight was nearly associated with an increased odds of PTD (1.46) for spontaneous delivery [30].

Maternal BMI and FA supplementation can affect the risk of PTD. Some studies have shown that there is an inverse interaction between BMI and serum folate levels [31, 32]. Distribution of folate in the body is significantly affected by obesity, and should pregnancy occur, it may reduce the amount of folate available to the developing embryo [33–35]. The FA supplement guideline in Canada suggests that women with a BMI >35 kg/m^2^ should take 5-mg folate supplements daily to prevent a poor outcome [36].

Whether there is any interactive effect between preconception BMI and FA supplementation for PTD is unclear. These previous studies mentioned above were mostly based on Caucasians, and there is a lack of Chinese population data. In this study, we aimed to determine whether FA supplementation can reduce the risk of PTD in China. We also investigated whether there is an interaction between FA supplementation and maternal BMI for the risk of PTD in the Chinese population.

## Methods

### Study design

This study was designed as a retrospective cohort study. From 2010, the free National Pre-pregnancy Checkups Project (NPCP) for rural women was carried out, which collected the largest pregnancy cohort data from the preconception stage in China. The NPCP is a population-based, free, preconception medical examination and services for rural reproductive-age couples who are trying to conceive in China. The NPCP covers couples who are preparing for pregnancy, from a preconception examination to pregnancy outcome follow-up. In 2010, the NPCP was first carried out in a 100-district region in China. In this study, examination data of the 100 districts in 2010 was used. The 100 districts are distributed throughout 18 provinces, including four provinces in eastern China (Jiangsu, Zhejiang, Shandong, and Guangdong), six provinces in central China (Hebei, Jilin, Anhui, Henan, Hubei, and Hunan), and eight provinces in western China (Guangxi, Chongqing, Sichuan, Guizhou, Yunnan, Shanxi, Gansu, and Xinjiang). The total population of the research area in 2010 was 7.16 million (females, 3.49 million; males, 3.67 million, approximately 5 % of the total population of China [37]). The rural population in the 100 districts was 4.76 million (66 % of the total population). Rural couples trying to conceive who lived in these areas could receive a free medical checkup in county-level medical institutions from January 2010 to December 2010 in the project. The clinical data were collected during the preconception medical examination. Information on socioeconomic background, reproductive history and history of illness, lifestyle behaviors, and dietary habits were carefully collected through face-to-face interviews by qualified nurses. Physical and biochemical examinations were also carried out by medical staff at the same time, including measurement of maternal height, weight, blood pressure, and hemoglobin levels.

After the examination, the couples received two follow-up interviews. The first interview was a telephone interview carried out by a trained nurse 3 months after conception. This interview was designed to obtain information of the last menstrual period and FA supplement use status (including the time when first taking FA). The second interview was carried out face-to-face or by telephone 1 month after delivery. Newborn information was collected by a trained interviewer according to the participants’ answers. The current study was conducted in accordance with the Declaration of Helsinki (2000) of the World Medical Association and the protocols were approved by the Institutional Research Review Board at the National Population and Family Planning Commission. Informed consent in Chinese was obtained from all NFPC participants.

### Study population

**Fig. 1:**
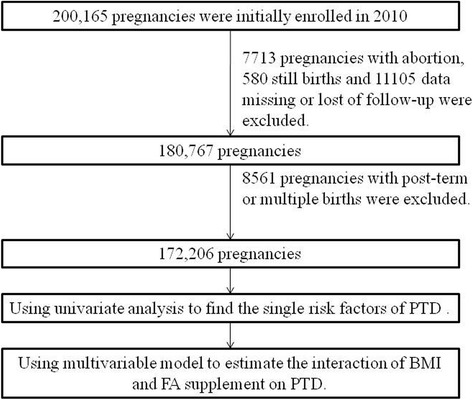
Flowchart of participant recruitment

Couples who attended the preconception medical examination and obtained successful conception within 6 months in the cohort were eligible (200,165). A total of 19,398 participants were excluded because of missing data, lost to follow-up, abortion (spontaneous or induced), and stillbirth. Women with post-term pregnancy (≥42 weeks) and multiple pregnancies were not included in the study (8561). Finally, 172,206 pregnancies were selected for analysis (Fig. 1).

### Definition of variables

The outcome variable in this analysis was PTD, which was defined as neonates who were born alive before 37 weeks of pregnancy were completed. Gestational age was calculated from the last menstrual period and the birth date. The independent variables were those expected to be associated with PTD based on current literature [38], including pertinent health variables (maternal preconception BMI, anemia, diabetes, and hypertension), pregnancy history, health behavior, and demographic variables. Preconception BMI (kg/m^2^) was calculated using maternal weight and height during the preconception physical examination. Because the participants in this study were all Chinese women, Chinese BMI classification standards were used in this study to classify different BMI groups. BMI <18.5 was considered underweight, BMI ≥18.5 and <24 was considered normal, BMI ≥24 and <28 was considered overweight, and BMI ≥28 was considered obese according to the Chinese population standards [39, 40].

FA supplementation was based on self-reported use in the first interview. FA supplementation was defined as women who had used supplements and had either taken FA alone or multivitamins (containing FA). FA supplement information was obtained in the first trimester follow-up. Therefore, we divided FA supplementation into three groups according to the time when the participants began to take FA: none, after conception, and preconception. Hypertension was defined as high blood pressure in the preconception examination (SBP ≥140 mmHg or DBP ≥90 mmHg) and self-reported hypertension. Diabetes was defined as self-reported diabetes or blood sugar ≥7 mmol/L. Anemia was defined as hemoglobin levels <110 g/L when the participant took the biochemical examination in the preconception service. Preconception dietary habits were defined as risk factors based on the self report, such as “dislike vegetables” and “vegetarian”, which indicated “seldom eat meat and eggs”. Tobacco use was defined as smoking at least one cigarette per day for at least 1 year in current smokers.

### Statistical analysis

All analyses were generated using SAS software (version 8.2, SAS Institute Inc., North Carolina). The general characteristics of mothers and children based on different categories of maternal preconception BMI and FA supplementation were compared using the chi-squared test and *t*-test. Logistic regression was used to estimate the crude odds ratio (cOR) and the adjusted odds ratio (aOR) with 95 % confidence intervals (CIs) of PTD [41]. Covariates in the multivariable models included demographic factors (maternal age, education level, ethnic group, and career) and preconception risk factors (hypertension, anemia, alcohol use). The time of FA supplement was calculated by setting the dummy variable. The effect of FA supplementation of different weight group was calculated through the contrasts estimated in the model. A significant *p* value was set at 0.05.

## Results

The mean age of the participants was 24.98 ± 3.87 years and the mean gestational age was 39.14 ± 1.67 weeks. The mean BMI was 21.03 ± 5.39 kg/m^2^. A total of 9573 infants were born PTD. 159,635 (92.7 %) mothers were 21–35 years old, 163,767 mothers were Han ethnic (95.1 %), and 163,423 had at least a junior or higher school education (94.9 %). The proportion of FA supplementation was 76.8 % and the proportion of an abnormal BMI (obese, overweight, and underweight) was 24.5 %. We compared maternal characteristics grouped by preconception BMI and FA supplementation status. Overweight or obese women were more likely to have hypertension or diabetes at preconception than women with a normal BMI or underweight women (Table 1). The proportion of women with high school or higher education experience was higher in women using FA than in nonusers. More population characteristics are shown in Table 1.

**Table 1:** Maternal characteristics in study population grouped by preconception body mass index and folic acid supplement

	Preconception body mass index(kg^2^)				*P*	Folic acid supplement			*P*
	<18.5	18.5-23.9	24-27.9	≥28		Never	1st trimester	Preconception	
Maternal characteristics									
No of subject	23429	129982	15880	2915		39871	41457	90878	
Demographic information (%)									
Maternal age (yr)	24.96(3.19)	24.18(3.82)	26.01(4.54)	26.60(4.70)	<0.001	24.85(3.94)	24.77(3.69)	25.13(3.90)	<0.001
Maternal education level									
Primary school	4.72	4.96	6.78	8.03	<0.001	5.01	4.51	5.50	<0.001
Junior school	60.56	67.38	71.79	72.42		73.50	63.63	65.57	
High school	21.82	18.81	15.29	14.68		15.35	20.68	19.51	
College and above	12.89	8.85	6.13	4.87		6.14	11.18	9.42	
House wife									
Yes	2.60	2.21	2.15	2.06	0.002	2.11	2.41	2.24	0.013
No	97.40	97.79	97.85	97.94		97.89	97.59	97.76	
Chinese Ethnic group									
Chinese Han	95.36	95.02	95.41	95.64	0.015	94.98	96.47	94.55	<0.001
Others	4.64	4.98	4.59	4.36		5.02	3.53	5.45	
Maternal risk factors (%)									
Previous preterm delivery									
No	99.75	99.83	99.62	99.59	<0.001	99.79	99.83	99.78	0.239
Yes	0.25	0.17	0.38	0.41		0.21	0.17	0.22	
Preconception hypertension									
No	98.41	97.99	95.74	90.05	<0.001	97.29	98.08	97.71	<0.001
Yes	1.59	2.01	4.26	9.95		2.71	1.92	2.29	
Anemia									
No	84.54	85.99	85.21	87.38	<0.001	83.98	83.41	87.58	<0.001
Yes	15.46	14.01	14.79	12.62		16.02	16.59	12.42	
Diabetes mellitus									
No	98.34	98.77	98.05	96.84	<0.001	98.63	98.28	98.76	<0.001
Yes	1.66	1.23	1.95	3.16		1.37	1.72	1.24	
Dislike vegetables									
No	99.24	99.37	99.17	99.11	0.004	99.27	99.34	99.34	0.286
Yes	0.76	0.63	0.83	0.89		0.73	0.66	0.66	
Preconception alcohol use									
No	96.79	97.01	97.20	97.32	0.080	97.43	96.83	96.89	<0.001
Yes	3.21	2.99	2.80	2.68		2.57	3.17	3.11	
Preconception Tobacco use									
No	99.63	99.70	99.57	99.31	<0.001	99.70	99.58	99.70	0.001
Yes	0.37	0.30	0.43	0.69		0.30	0.42	0.30	
Preterm delivery									
No	94.11	94.57	94.05	93.69	0.001	92.08	94.98	95.23	<0.001
Yes	5.89	5.43	5.95	6.31		7.92	5.02	4.77	

Women with an abnormal BMI had an increased risk of PTD (OR = 1.09, OR = 1.10, and OR = 1.17 for underweight, overweight, and obese, respectively). Preconception disease also raised the risk of PTD (OR = 1.49 and OR = 1.19 for hypertension and anemia, respectively). Women using alcohol had a higher risk of PTD (OR = 1.24). The risk of PTD was decreased by taking FA supplements, and this phenomenon was time-related. Taking FA supplements in the preconception period or in the first trimester reduced the risk of PTD (OR = 0.58 and OR = 0.61, respectively). The difference between the preconception period and the first trimester was not significant by transforming the first trimester as the reference group (*p =* 0.053). Details were listed in Table 2.

**Table 2:** Crude odds ratio (95 % confidence intervals) of preterm delivery by maternal characteristics and single risk factor

	Number	PTD		
		%	cOR (95 % CI)	*P* value
Maternal characteristics				
Maternal age				
≤ 20 years	8844	5.89	1.07 (0.98,1.17)	0.14
21–35 years	159640	5.53	1	
> 35 years	3722	6.18	1.13 (0.98,1.29)	0.09
Maternal education level				
Primary school and below	8866	4.87		
Junior school	115279	6.02	1.25 (1.13,1.38)	<0.01*
High school	32418	4.42	0.90 (0.81,1.01)	0.07
College and above	15643	4.88	1 (0.89,1.13)	0.97
Maternal career				
House wife	3878	7.01	1	
Others	168328	5.53	1.29 (1.14,1.46)	<0.01*
Chinese Ethnic group				
Chinese Han	163786	5.67	1	
Others	8420	3.46	0.60 (0.53,0.67)	<0.01*
Maternal risk factors				
Previous preterm delivery				
No	171852	5.56	1	
Yes	354	6.78	1.24 (0.82,1.87)	0.32
Preconception BMI				
Underweight	23429	5.89	1.09 (1.03,1.16)	<0.01*
Normal weight	129982	5.43	1	
Overweight	15880	5.95	1.10 (1.03,1.18)	0.01*
Obese	2915	6.31	1.17 (1.01,1.36)	0.04*
Preconception hypertension				
No	168248	5.5	1	
Yes	3958	7.98	1.49 (1.33,1.67)	<0.01*
Anemia				
No	147657	5.42	1	
Yes	24549	6.37	1.19 (1.12,1.25)	<0.01*
Diabetes mellitus				
No	169819	5.55	1	
Yes	2387	6.12	1.11 (0.94,1.31)	0.23
Folic acid supplement				
Never	39871	7.92	1	
1st trimester	41457	5.02	0.61(0.58,0.65)	<0.01*
Preconception	90878	4.77	0.58(0.56,0.61)	<0.01*
Dislike vegetables				
No	171047	5.55	1	
Yes	1159	6.47	1.18(0.93,1.49)	0.17
Preconception alcohol use				
No	167040	5.52	1	
Yes	5166	6.78	1.24 (1.11,1.39)	<0.01*
Preconception tobacco use				
No	171641	5.56	1	
Yes	565	6.19	0.85 (0.58,1.25)	0.42

*Note*: cOR = crude odds ratio

An interaction effect was found between preconception BMI and FA supplementation for PTD (Table 3). Taking FA supplements reduced the risk of PTD in all groups. Non-users with a normal BMI had a baseline risk of 7.95 PTD births per 100 live births, whereas mothers who took FA supplements had 4.69 PTD births per 100 live births (OR = 0.60). The OR of FA supplementation varied in different BMI groups. In underweight group, the OR of FA was 0.57 (95 % CI: 0.51, 0.64); in overweight group, the OR was 0.85 (95 % CI: 0.73,0.98); in obese group, the OR was 0.77 (95 % CI: 0.65,0.91). The interaction between maternal BMI and time of FA supplements was compared to examine why the protective effect was different among different BMI groups. We found that the protective effect of FA supplement in women with normal or underweight had similar values. The effect of FA supplement was different in overweight and obesity group, especially in preconception. Women with a BMI <24 kg/m^2^ (including normal and underweight groups) who took FA supplements from preconception had a lower risk for PTD than in the first trimester (OR: 0.58 vs. 0.65 in the normal weight group, 0.56 vs. 0.60 in the underweight group). Women with a BMI ≥24 kg/m^2^ (including the overweight and obese groups) who took FA supplements from the first trimester had a lower risk of PTD than at preconception (OR: 0.65 vs. 0.94 in the overweight group, 0.60 vs. 1.15 in the obesity group). Compared with no takers, obese women took FA supplement in preconception had an OR of 1.15 (not statistical significant).

**Table 3:** Effect of FA supplement in different BMI group for PTD

BMI group	FA supplement OR (95 % CI)						
	No	Yes		1st trimester		Preconception	
Normal	ref	0.60	(0.57,0.63)	0.65	(0.61,0.69)	0.58	(0.55,0.62)
Underweight	ref	0.57	(0.51,0.64)	0.60	(0.52,0.69)	0.56	(0.49,0.63)
Overweight	ref	0.85	(0.73,0.98)	0.65	(0.53,0.79)	0.94	(0.80,1.10)
Obesity	ref	0.77	(0.65,0.91)	0.60	(0.37,0.97)	1.15	(0.81,1.64)

*Note*: Adjusted for maternal education, career, ethnic, hypertension, anemia, alcohol use

## Discussion

In our research population, the proportion of FA supplementation was as high as 76.8 % and more than 50 % women began to take FA supplements before conception. The reason for the high rate of supplementation in this population was based on a nation-wide free FA supplementation program, which was implemented in rural areas of China since 2009. The Chinese guideline of FA supplements is 0.4 mg/d from preconception to the end of the first trimester. Women who had rural household registration and planned to become pregnant were eligible to obtain FA tablets containing 0.4 mg FA for 6 months at no charge [42].

A negative association was found between FA supplementation and the risk of PTD in our population. FA supplementation showed a strong protective effect on PTD. This protective rate varied from 35 % to 40 % according to different supplementation times. Women who began to take FA supplements in the preconception period had the lowest risk of PTD (cOR = 0.58, details in Table 2). Similar results were reported in the USA, where the duration of preconceptional folate supplementation affected the protective effect for PTD. In a meta-analysis, many observational studies suggested a slight reduction in PTD, which was not consistent with the results from randomized, controlled trials [43]. The risk of spontaneous preterm birth decreased with the duration of preconceptional folate supplementation, and taking FA supplements over 1 year could reduce the risk of early spontaneous preterm birth by 50–70 %. In contrast, a cohort of Norwegians showed the opposite result, where preconceptional FA supplementation starting more than 8 weeks before conception was associated with an increased risk of PTD (hazard ratio = 1.19) [44]. Another study showed a 20 % lowered risk of PTD was found in the first trimester compared with preconception [33].

In our population, we found that women who were underweight/overweight/obese were at increased risk of PTD in single risk factor analysis (Table 2). Similar results were also reported in the USA and India [29, 45, 46]. Spontaneous preterm birth could be caused by maternal thinness associated with decreased blood volume and fewer vitamins and minerals, which are associated with increased maternal infections. Serum folate levels are lower in obese women than in normal or underweight women [32]. Obese women are more likely to develop pre-eclampsia and diabetes, and indicated preterm births are associated with these disorders [42]. In this study, we could only compare the interaction between FA supplementation and maternal BMI. The direct factor (folate levels) in the population was not compared (without original data). FA supplementation can reduce homocysteine, which causes pre-eclampsia and affects placental function and fetal maturity, eventually leading to PTD. Therefore, analyzing the interaction of FA and BMI for prevention of PTD is important.

An interaction between FA supplements and maternal BMI was found in our population, and the protective effect of FA varied in different BMI groups. Taking FA supplements of 0.4 mg/d (regardless of when they started) reduced the risk of PTD in all of the BMI groups. The time of taking FA supplement was very important in women with abnormal BMI. In the underweight group, there was very little difference between 1st trimester and preconception. But in overweight and obesity group, the difference was very large. We hypothesize that maternal body size is associated with folate metabolism, which can influence maternal homocysteine levels and affect embryonic development and gestational weeks [23, 47]. Distribution of folate in the body is significantly affected by BMI, and obesity might reduce the amount of folate available to the developing embryo [35, 36]. Obesity-associated metabolic alterations could have an effect on folate use or increase folate requirements [48, 49]. RBC folate increased incrementally with BMI, while serum folate concentrations were lower in obese groups [50]. We conclude that FA supplementation and maternal obesity can influence homocysteine levels, which might affect placental function and eventually lead to PTD. The distribution of folate might be changed in pregnancy period. We also compared the interaction between the time of FA supplements and maternal BMI. Women with a BMI <24 kg/m^2^ (including the normal and underweight groups) who took FA supplements from preconception had a lower risk for PTD than in the first trimester. Women with a BMI ≥24 kg/m^2^ (including the overweight and obese groups) who took FA supplements from the first trimester had a lower risk of PTD than at preconception. Several studies have shown that different supplementation times can change the protective effect of FA [9, 51]. In a similar study on NTD, the protective of FA was also weaker in the overweight and obesity population than in the normal and underweight population [52]. We suggest that a diverse plan of FA supplementation should be carried out according to women’s BMI category. The time and dose of FA supplement for obesity should be taken into account.

## Limitations

The primary limitation in the present study was misclassification of FA supplemental status. We relied on the self-reported use of FA supplements, which could lead to misclassification of FA supplemental status. We compared the proportion of FA supplements in our study with other studies (77 % vs. 68–92 % [47, 53]). The name and content of the FA supplement was not investigated in the first trimester interview. Therefore, we could not separate FA supplements and multiple vitamin supplements, including FA. Dietary folate was also not estimated in this cohort. We could not calculate the exact folate level. Further research should be carried out to verify the results of this study.

The second limitation of this study was that PTD could not be divided into indicated and spontaneous PTD. Pre-pregnancy obesity is associated with a higher risk of indicated, but not spontaneous, PTD [30]. This study was a retrospective cohort study. We could not analyze the risk of iatrogenic PTD because this type of PTD was not classified in the follow-up records. Detailed data should be collected to estimate the protective effect of FA between indicated PTD and spontaneous PTD in the future.

Because of the uncertainty of natural conception, we selected women who became pregnant in the following 6 months after the preconception medical examination as our research population. All statistical research was carried out in this population, which could also have reduced the effect of a change in maternal medical status on the results. Reported supplementation of FA for 1 year prior to conception has been linked with a decreased risk of preterm birth [54]. In our cohort, we did not test the protective effect of FA from 1 year before conception in our population. Because our study design only included 6 months of FA supplements in the pregnant population, the results may not apply to the whole population.

## Conclusions

In our study, FA supplements reduced the risk of PTD, while abnormal BMI raised the risk of PTD, although higher BMI categories did not have this higher risk once adjusted analysis was conducted. The protective effect of FA supplementation for PTD is reduced in women whose BMI was equal or greater than 24 kg/m^2^. To get better protection of FA supplementation, women with BMI < 24 kg/m^2^ should begin to use in preconception, while women with BMI ≥24 kg/m^2^ should begin to use after conception.

## Abbreviations

FA: Folic acid
BMI: Body mass index
PTD: Preterm delivery
cOR: Crude odds ratio
aOR: Adjusted odds ratio
CIs: Confidence intervals
